# Incidence of inpatient venous thromboembolism in treated patients with rheumatoid arthritis and the association with switching biologic or targeted synthetic disease-modifying antirheumatic drugs (DMARDs) in the real-world setting

**DOI:** 10.1136/rmdopen-2019-001013

**Published:** 2019-09-23

**Authors:** Huifang Liang, Raghava Danwada, Dianlin Guo, Jeffrey R Curtis, Ryan D Kilpatrick, Barbara Hendrickson, Syed S Islam

**Affiliations:** 1 Global Epidemiology, Pharmacovigilance and Patient Safety, AbbVie Inc, North Chicago, Illinois, USA; 2 Division of Clinical Immunology and Rheumatology, University of Alabama at Birmingham, Birmingham, Alabama, USA; 3 Pharmacovigilance and Patient Safety, AbbVie Inc, North Chicago, Illinois, USA

**Keywords:** rheumatoid arthritis, disease-modifying antirheumatic drugs (DMARDs), cardiovascular disease, epidemiology

## Abstract

**Objectives:**

To assess incidence rates (IRs) of VTE in patients with rheumatoid arthritis (RA) on different DMARDs and DMARD switchers.

**Methods:**

Adults with RA on a DMARD between 2007 and 2017 were studied in a US claims database. Conventional synthetic DMARD (csDMARD) users, first biologic/targeted synthetic DMARD (b/tsDMARD) users and b/tsDMARD switchers (from a b/tsDMARD to another b/tsDMARD) were followed for inpatient VTE (pulmonary embolism (PE)/deep vein thrombosis (DVT)). Crude and adjusted IR and 95% CIs of VTE were estimated. HRs for VTE were estimated via Cox regression. VTE risk was also evaluated by number of switches between b/tsDMARDs and in patients without a VTE history.

**Results:**

The age and sex standardised IR (95% CI) of VTE (per 100 person-years) was 0.86 (0.70 to 1.03), 0.60 (0.52 to 0.68) and 0.58 (0.51 to 0.65) for b/tsDMARD switchers, first b/tsDMARD users and csDMARD users, respectively. After adjustment, b/tsDMARD switchers had an increased risk of VTE, compared with csDMARD users, HR_adj_ (95% CI) being 1.36 (1.16 to 1.58), 1.36 (1.13 to 1.63) and 1.47 (1.18 to 1.83) for VTE, DVT and PE, respectively. Compared with first b/tsDMARD users, the HR_adj_ (95% CI) for VTE was 1.35 (1.15 to 1.60) for first b/tsDMARD switchers and 1.48 (1.19 to 1.85) for second b/tsDMARD switchers.

**Conclusions:**

In RA, b/tsDMARD switchers have a higher VTE risk compared with csDMARD users and first b/tsDMARD users. Switching b/tsDMARDs may be a proxy for higher disease severity or poorly controlled RA and an important confounder to consider in obtaining unbiased estimates of VTE risk in observational RA safety studies.

**Key messages**

**What is already known about this subject?**

- The risk of venous thromboembolism (VTE) is increased in patients with rheumatoid arthritis (RA).

**What does this study add?**

- After controlling for multiple risk factors, patients with RA who switch biologic/targeted synthetic disease-modifying antirheumatic drugs (b/tsDMARDs) have a higher VTE risk compared with conventional synthetic DMARD users and first b/tsDMARD users. This risk was even higher in the subgroup who switched b/tsDMARDs twice.
- Switching may be a proxy for higher disease severity or poorly controlled RA and an important confounder to consider in obtaining unbiased estimates of VTE risk in observational RA safety studies.

**How might this impact on clinical practice?**

- Drug safety studies may want to consider treatment switching as a potential proxy for unmeasured clinical RA disease activity and severity which may confound associations with important clinical outcomes such as VTE.

## Introduction

Venous thromboembolism (VTE), including deep vein thrombosis (DVT) and pulmonary embolism (PE), is the third most common cardiovascular disease after myocardial infarction (MI) and stroke.^1^ In the general population, the risk of VTE is approximately 0.1–0.4 per 100 person-years.^1–3^ Recent studies consistently showed that rheumatoid arthritis (RA) is associated with a 1.5-fold to 1.6-fold increased risk of VTE compared with the general population.^3–8^ The increased risk in RA is believed to be attributable in part to proinflammatory cytokine production, oxidative stress and endothelial dysfunction.^9 10^ Systemic inflammation modulates thrombotic responses by suppressing fibrinolysis, upregulating procoagulant and downregulating anticoagulants.^11^

Patients with RA may be initially treated with conventional synthetic disease-modifying antirheumatic drugs (csDMARDs).^12^ Patients with moderate to severe RA who do not respond to csDMARDs may switch to biologic or targeted synthetic DMARDs (b/tsDMARDs), which may be administered with or without csDMARDs. The switch from csDMARD to b/tsDMARDs or from one b/tsDMARD to another b/tsDMARD may indicate inability to control systemic inflammation, which may predispose these patients with RA to a higher risk of VTE. Patients who are bDMARD users have been found to have more severe RA (assessed by treatment) than patients treated with csDMARDs.^13 14^

In addition to RA, higher risk of VTE has also been associated with increasing age,^15 16^ male sex,^17–19^ African-American race,^16^ alcohol abuse,^20^ drug abuse, smoking,^21 22^ a history of prior VTE,^23^ cardiovascular risk factors (obesity, hypertension, diabetes mellitus, hyperlipidemia, congestive heart failure (CHF), MI and stroke),^24^ hospitalisation and recent surgery,^25^ chronic kidney disease (CKD),^26^ any malignancy,^27^ serious infections,^28 29^ chronic obstructive pulmonary disease (COPD),^15 30^ asthma,^31 32^ viral hepatitis,^33^ diverticular disease,^34^ gastroduodenal ulcer,^35^ inflammatory bowel disease,^36^ depression, and major knee and hip surgeries.^36 37^

Limited data are available on risk of VTE in DMARD users. Kim *et al* reported the incidence rates (IRs) of hospitalised VTE in csDMARD initiators and bDMARD initiators.^6^ Weinblatt *et al* recently reported the IR of VTE in patients with RA treated with baricitinib, a tsDMARD.^38^ Beyond reporting only overall rates of VTE, b/tsDMARD switching in real-world data that may be proxies for higher RA disease severity and more refractory patients who may be at greater risk of VTE are important to identify. This information would potentially increase the validity of future observational drug safety studies. To this end, DMARD-treated patients in this analysis were characterised by line of therapy and further, by whether and how many times they switched from a b/tsDMARD to another b/tsDMARD. We evaluated whether VTE rates could be increased according to line of RA therapy and b/tsDMARD switching, which may be a useful proxy for higher disease severity or refractory RA.

## Methods

### Study design and data source

A cohort study was conducted using the Optum Clinformatics Data Mart, a US claims database containing anonymised longitudinal data for patients insured with the United HealthCare plans since 2000.

### Study population

Adults were eligible for the study if they had at least two diagnoses of RA (see online supplemental data) from medical claims ≥7 days apart in 2007–2017, received at least one systemic DMARD after the first RA diagnosis in 2007–2017 and had at least 1 year of continuous health plan enrolment before the index DMARD treatment, with a gap of ≤45 days allowed. The index date was defined as the first date of DMARD treatment after the first RA diagnosis in the study period. No exclusion criteria were applied.

10.1136/rmdopen-2019-001013.supp1 Supplementary data

### DMARD treatment

**Figure 1:**
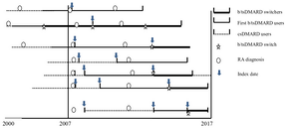
Identification of biologic/targeted synthetic disease-modifying antirheumatic drug (b/tsDMARD) switchers, first b/tsDMARD users and conventional synthetic DMARD users during the study period. Note: the first treatment after the diagnosis of rheumatoid arthritis that met the definition of treatment group and during the study period (2007–2017) was used to define the treatment group.

Figure 1 presents a diagram to identify three cohorts; some patients might be in multiple cohorts. During the study period, those who discontinued one or more previous b/tsDMARDs (see online supplemental data for generic names) and switched to another b/tsDMARD were defined as b/tsDMARD switchers. Switching from intravenous administration to subcutaneous administration of the same drug or vice versa was not considered a switch. First b/tsDMARD users were those on their first observed b/tsDMARD therapy. Both b/tsDMARD switchers and first b/tsDMARD users may be treated with csDMARD previously and concomitantly. csDMARD users were those who received csDMARD but had not received any b/tsDMARDs. Since our objective was not to evaluate the effect of RA therapy (together or individually) but to assess the ability of switching among b/tsDMARDs to serve as a proxy for uncontrolled disease activity on VTE risk, individual drugs were not assessed in the primary analysis. Others have discussed or explored differences in VTE rates in those treated with tsDMARDs.^39 40^ Therefore, as a secondary analysis, the incidence rate of VTE was also explored by drug group (TNF-α inhibitors, non-TNF-α biologics, tsDMARD tofacitinib and biosimilars).

### Outcomes and covariates

The VTE outcome was defined as the first hospitalised DVT or PE during follow-up, while when analysed separately, the earliest hospitalised DVT during follow-up and the earliest hospitalised PE during follow-up.^6 41 42^ Data on patient characteristics (age, sex, race, calendar year and geographical region), behavioural factors (smoking, alcohol/drug abuse), hospitalisation within 1 year before index date, cardiovascular risk factors, comorbidities/medical histories, history of dispensed non-steroidal anti-inflammatory drugs (NSAIDs, including Cox-2 inhibitors), corticosteroids and anticoagulants any time before the cohort entry were also included as covariates. For medical history or comorbidities, we required two outpatient diagnoses on different dates or one inpatient diagnosis. Hospitalisation was required for serious infections. Although some patients may have contributed to different treatment cohorts, the time windows for their baseline characteristics and outcome ascertainment were different.

## Statistical analyses

Descriptive statistics were generated for baseline patient characteristics. A standardised difference with an absolute value of ≥0.1 was used to indicate meaningful difference between groups at baseline.^43^ The follow-up started from the day after the index date to the earliest of an outcome event, disenrolment from the healthcare plan, treatment discontinuation, death while hospitalised or 30 September 2017. The treatment time window was defined from the index date through the last drug dose plus an exposure window of 90 days for b/tsDMARDs^44^ and 37 days for csDMARD, based on a 30-day discontinuation gap for methotrexate^45^ plus 7 days of prefilling a prescription. The on-treatment approach was used to assess the IR of VTE with justifications (online supplemental data). The 1-year cumulative incidence was estimated using the number of inpatient VTE cases within 1 year after the index date divided by the number of patients. The number needed to harm (NNH) was calculated by dividing 1 by the absolute risk increase for b/tsDMARD switchers and first b/tsDMARD users, respectively, with csDMARD users as the reference group.

For csDMARD users, the follow-up ended at the time of initiating a b/tsDMARD; for first b/tsDMARD users, the follow-up ended at the time of switching to a second b/tsDMARD. The follow-up of b/tsDMARD switchers continued when they switched to other b/tsDMARDS. In the main analysis, no further distinction was made among b/tsDMARD switchers (ie, all b/tsDMARD switchers were represented as a single exposure group). However, in a sensitivity analysis, b/tsDMARD switchers were further classified into ‘first’ b/tsDMARD switchers (of note, some ‘first’ b/tsDMARD switchers may have switched before the index date) and ‘second’ b/tsDMARD switchers after the index date, with the time of switch to a different b/tsDMARD treated as a censoring event for VTE for both cohorts. Eligibility criteria were re-assessed and covariates were updated for ‘second’ b/tsDMARD switchers based on the second switch date. Due to significant reduction in number of patients, risks beyond the ‘second’ b/tsDMARD switchers were not assessed.

The crude IR of first hospitalised VTE, DVT and PE occurring after the start of follow-up and 95% CIs^46^ were estimated by treatment groups and selected patient characteristics. Standardised IRs were calculated for three treatment groups, and subgroup of drugs among biologic users, applying age and sex-specific IRs to population age and sex distribution from US Census 2010 in a direct method,^47^ with 95% CI estimated using normal approximation.^48^

Cox proportional-hazards models were used to estimate the effect (HRs) of treatment on the risk of VTE outcomes, with primary model adjusted for demographics. Additional variable selection was based on clinical knowledge and F statistic from Cox models. Data management and statistical analyses were performed using SAS V.9.4 (SAS Institute, Cary, North Carolina, USA). Institutional review board review is exempt for this study as data were de-identified.

## Results

**Figure 2:**
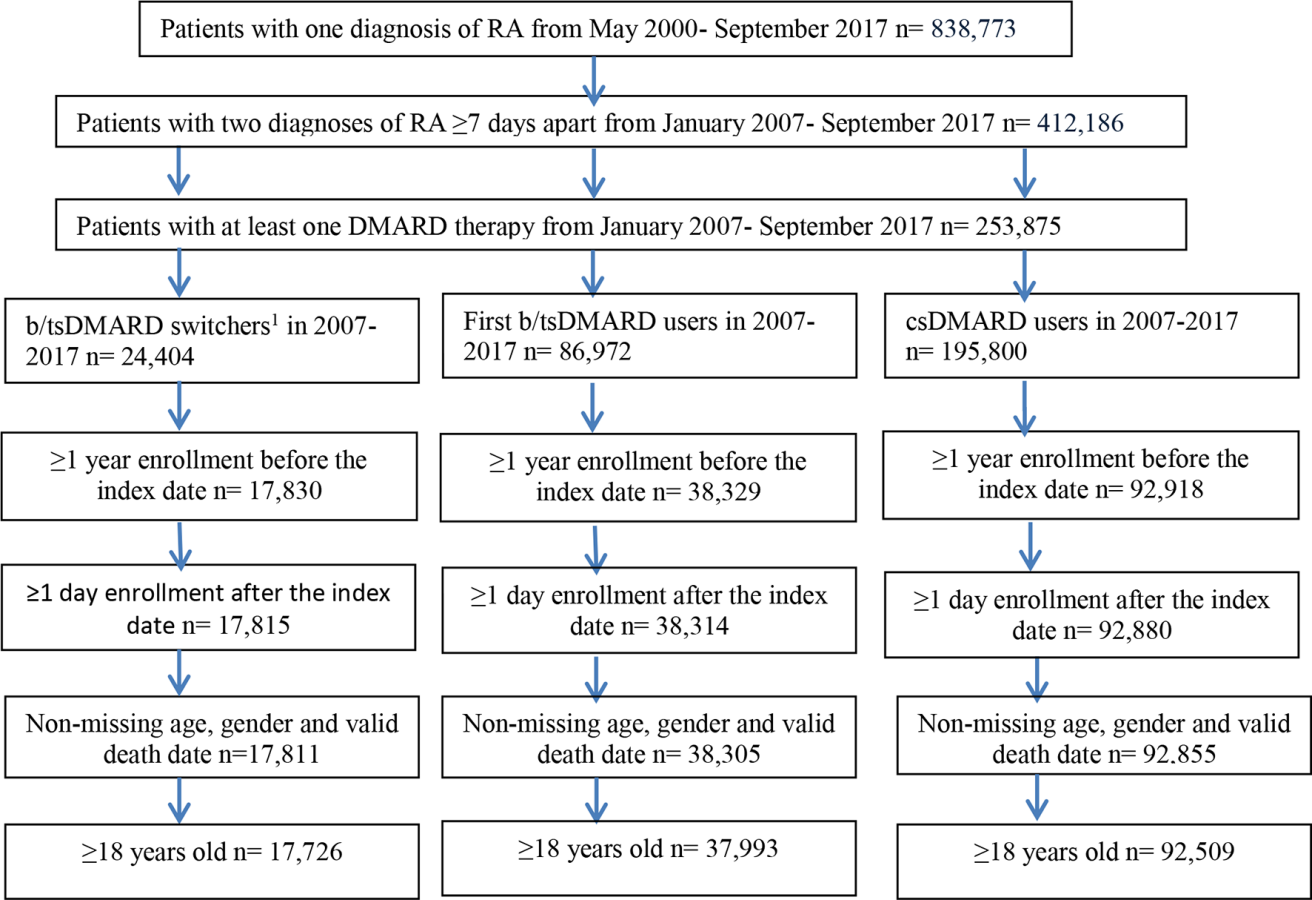
Patient attrition in patients with rheumatoid arthritis in the Optum Cliniformatics Data Mart from May 2000 through September 2017.

Patient disposition is presented in figure 2. Table 1 presents patient characteristics for b/tsDMARD switchers, first b/tsDMARD users and csDMARD users. Each cohort had an average enrolment period of 4 years or more before the index date. A total of 14 823 (16.0% of 92 509) csDMARD users became first b/tsDMARD users, and 9757 (25.7% of 37 993) first b/tsDMARD users became b/tsDMARD switchers (data not shown). b/tsDMARD users were on average 5.5 years younger than csDMARD users. The most common disease history or comorbidities in the study population were hypertension (>40%) and hyperlipidemia (~40%), followed by diabetes mellitus (>15%), serious infections (>10%), CKD, COPD, asthma, depression (~10%), inflammatory bowel disease, malignancy and MI/stroke (<10%). Compared with csDMARD users, first b/tsDMARD users had a lower proportion of hypertension, hyperlipidemia, diabetes mellitus, CHF, atrial fibrillation or flutter, and MI/stroke. b/tsDMARD switchers had a higher proportion of patients with a history of serious infections, major knee and hip surgeries, and corticosteroid use than first b/tsDMARD users and csDMARD users. No differences were identified between groups for behavioural risk factors and inpatient hospital stays (online supplemental table S1).

**Table 1:** Demographics and baseline characteristics of patients with rheumatoid arthritis on DMARD therapy

	b/tsDMARD switchers (1)	First b/tsDMARD users (2)	csDMARD users (3)	Standardised difference 1 vs 3	Standardised difference 2 vs 3	Standardised difference 1 vs 2
N	17 726 (100.0)	37 993 (100.0)	92 509 (100.0)	–	–	–
Sex						
Female	13 948 (78.7)	28 489 (75.0)	70 014 (75.7)	0.072	−0.016	0.088
Male	3778 (21.3)	9504 (25.0)	22 495 (24.3)	−0.072	0.016	−0.088
Age (years, mean±SD)	54.6±12.8	54.7±13.4	60.1±14.6	−**0.401**	−**0.385**	−0.003
Race						
White	12 432 (70.1)	26 316 (69.3)	61 345 (66.3)	0.082	0.064	0.017
Black	1483 (8.4)	3208 (8.4)	9101 (9.8)	−0.049	−0.049	0.000
Hispanic	1915 (10.8)	4196 (11.0)	11 160 (12.1)	−0.041	−0.034	−0.006
Asian	390 (2.2)	925 (2.4)	2315 (2.5)	−0.020	−0.006	−0.013
Unknown	1508 (8.5)	3348 (8.8)	8588 (9.3)	−0.028	−0.017	−0.011
Pre-enrolment (years, mean±SD)	4.4±3.2	4.0±2.9	4.3±3.1	0.032	−**0.100**	**0.110**
History of alcohol abuse/drug abuse	3551 (20.0)	6494 (17.1)	15 714 (17.0)	0.077	0.003	0.075
Ever smoker	2896 (16.3)	5414 (14.2)	12 902 (13.9)	0.067	0.009	0.058
Medical history						
VTE	615 (3.5)	1070 (2.8)	3191 (3.4)	0.005	−0.035	0.040
DVT	476 (2.7)	838 (2.2)	2541 (2.7)	0.000	−0.032	0.032
PE	261 (1.5)	427 (1.1)	1247 (1.3)	0.017	−0.018	0.035
Hypertension	8235 (46.5)	15 925 (41.9)	46 803 (50.6)	−0.082	−**0.175**	0.093
Diabetes mellitus	3028 (17.1)	5706 (15.0)	17 478 (18.9)	−0.047	−**0.104**	0.057
Hyperlipidemia	7082 (40.0)	13 520 (35.6)	40 467 (43.7)	−0.075	−**0.166**	0.091
Congestive heart failure	708 (4.0)	1268 (3.3)	5410 (5.8)	−0.083	−**0.120**	0.037
MI or stroke	1333 (7.5)	2452 (6.5)	8992 (9.7)	−0.079	−**0.117**	0.039
Atrial fibrillation/flutter	665 (3.8)	1171 (3.1)	4916 (5.3)	−0.072	**−0.110**	0.038
Chronic kidney disease	2021 (11.4)	3844 (10.1)	13 239 (14.3)	−0.087	−**0.129**	0.042
Malignancy	920 (5.2)	1799 (4.7)	6348 (6.9)	−0.071	−0.094	0.023
Serious infections	2995 (16.9)	4955 (13.0)	13 354 (14.4)	0.069	−0.041	**0.110**
COPD	2049 (11.6)	3747 (9.9)	12 043 (13.0)	−0.043	−0.097	0.055
Asthma	2162 (12.2)	3692 (9.7)	9844 (10.6)	0.050	−0.030	0.080
IBD	1001 (5.7)	1883 (5.0)	3749 (4.0)	0.079	0.048	0.031
Depression	2146 (12.1)	3436 (9.0)	8618 (9.3)	0.091	−0.010	**0.101**
Major knee and hip surgeries	1253 (7.1)	803 (2.1)	331 (0.4)	**0.358**	**0.153**	**0.240**
Index drug						
Anti-TNF-α	11 946 (67.4)	33 617 (88.5)	–	–	–	−**0.526**
Non-TNF inhibitors	4933 (27.8)	3696 (9.7)	–	–	–	**0.477**
Tofacitinib	847 (4.8)	680 (1.8)	–	–	–	**0.169**
History of medication use						
NSAID	13 781 (77.7)	27 977 (73.6)	67 297 (72.7)	**0.116**	0.020	0.096
Cox-2 inhibitors	4443 (25.1)	8579 (22.6)	17 078 (18.5)	**0.160**	**0.102**	0.059
Systemic corticosteroids	6191 (34.9)	10 525 (27.7)	21 749 (23.5)	**0.253**	**0.096**	**0.156**
Anticoagulants	2684 (15.1)	4552 (12.0)	13 334 (14.4)	0.020	−0.071	0.091

Data are presented as n (%), unless otherwise specified. A standardised difference with an absolute value of greater than 0.10 indicates statistical significance for groups under comparison. With absolute values greater than 0.1, bolded values for standardised dfiference in the table indicates statistical significance.

anti-TNF, anti-tumour necrosis factor; b/tsDMARD, biologic or targeted synthetic DMARD; COPD, chronic obstructive pulmonary disease; csDMARD, conventional synthetic DMARD;DMARD, disease-modifying antirheumatic drug; DVT, deep vein thrombosis; IBD, inflammatory bowel disease;MI, myocardial infarction; NSAID, non-steroidal anti-inflammatory drug; PE, pulmonary embolism; VTE, venous thromboembolism.

Crude IRs of VTE, DVT and PE stratified by treatment groups and patient characteristics are presented in table 2. The overall crude IR (95% CI) of VTE was 0.79 (0.75 to 0.83) per 100 patient-years(PY). Among those who did not use anticoagulants in 1 year before index date, the IR of VTE was 0.62 (0.58 to 0.66) per 100 patient-years. The stratified analysis demonstrated that increased risk of VTE was associated with b/tsDMARD switching, male sex, increasing age, white and African-American races, behaviour factors, medical histories and history of medication use including dispensed anticoagulant and corticosteroid. NSAID use was not associated with an increased risk of VTE (online supplemental table S2).

**Table 2:** Incidence rate of VTE, DVT and PE per 100 patient-years by treatments, demographics and baseline characteristics in patients with rheumatoid arthritis

		Inpatient VTE		Inpatient DVT		Inpatient PE	
	N	Cases/PY	IR (95% CI)	Cases/PY	IR (95% CI)	Cases/PY	IR (95% CI)
All	148 228	1295/164 476.8	0.79 (0.75 to 0.83)	936/164 742.2	0.57 (0.53 to 0.61)	596/165 047.5	0.36 (0.33 to 0.39)
Treatment							
b/tsDMARD switchers	17 726	229/26 715	0.86 (0.75 to 0.98)	164/26 762.9	0.61 (0.52 to 0.71)	115/26 849.2	0.43 (0.35 to 0.51)
First b/tsDMARD users	37 993	364/54 138.1	0.67 (0.61 to 0.75)	263/54 215.9	0.49 (0.43 to 0.55)	165/54 297.2	0.30 (0.26 to 0.35)
csDMARDs	92 509	702/83 623.8	0.84 (0.78 to 0.90)	509/83 763.5	0.61 (0.56 to 0.66)	316/83 901	0.38 (0.34 to 0.42)
Demographics							
Sex							
Female	112 451	919/123 499.6	0.74 (0.70 to 0.79)	668/123 671.8	0.54 (0.50 to 0.58)	423/123 916.9	0.34 (0.31 to 0.38)
Male	35 777	376/40 977.2	0.92 (0.83 to 1.02)	268/41 070.4	0.65 (0.58 to 0.74)	173/41 130.6	0.42 (0.36 to 0.49)
Age (years)							
18–44	25 783	94/25 570.5	0.37 (0.30 to 0.45)	76/25 585.6	0.30 (0.23 to 0.37)	42/25 613.7	0.16 (0.12 to 0.22)
45–64	72 484	486/84 279.3	0.58 (0.53 to 0.63)	337/84 398	0.40 (0.36 to 0.44)	240/84 506.1	0.28 (0.25 to 0.32)
65–74	29 907	389/33 355.2	1.17 (1.05 to 1.29)	274/33 442.2	0.82 (0.73 to 0.92)	178/33 541.4	0.53 (0.46 to 0.61)
75+	20 054	326/21 271.8	1.53 (1.37 to 1.71)	249/21 316.5	1.17 (1.03 to 1.32)	136/21 386.3	0.64 (0.53 to 0.75)
Race							
White	100 091	955/117 943.6	0.81 (0.76 to 0.86)	694/118 146.7	0.59 (0.54 to 0.63)	443/118 360.6	0.37 (0.34 to 0.41)
Black	13 792	135/13 991.2	0.96 (0.81 to 1.14)	93/14 016.4	0.66 (0.54 to 0.81)	60/14 078.7	0.43 (0.33 to 0.55)
Hispanic	17 271	99/16 448.4	0.60 (0.49 to 0.73)	75/16 464.3	0.46 (0.36 to 0.57)	38/16 478.3	0.23 (0.16 to 0.32)
Asian	3630	20/3883.4	0.52 (0.31 to 0.80)	10/3887.4	0.26 (0.12 to 0.47)	12/3891.4	0.31 (0.16 to 0.54)
Unknown	6464	86/12 210.3	0.70 (0.56 to 0.87)	64/12 227.3	0.52 (0.40 to 0.67)	43/12 238.6	0.35 (0.25 to 0.47)
Medical history							
VTE							
Yes	4876	284/4342.6	6.54 (5.80 to 7.35)	207/4414.9	4.69 (4.07 to 5.37)	113/4472.1	2.53 (2.08 to 3.04)
No	143 352	1011/160 134.3	0.63 (0.59 to 0.67)	729/160 327.3	0.45 (0.42 to 0.49)	483/160 575.4	0.30 (0.27 to 0.33)
Medication use history							
Anticoagulants							
Yes	11 660	345/11 466.1	3.01 (2.7 to 3.34)	247/11 562.3	2.14 (1.88 to 2.42)	143/11 616	1.23 (1.04 to 1.45)
No	136 568	950/153 010.8	0.62 (0.58 to 0.66)	689/153 179.9	0.45 (0.42 to 0.48)	453/153 431.5	0.30 (0.27 to 0.32)
VTE and anticoagulants							
Yes	2945	224/2571.9	8.71 (7.61 to 9.93)	161/2639.5	6.10 (5.19 to 7.12)	90/2673.9	3.37 (2.71 to 4.14)
No	145 283	1071/161 905	0.66 (0.62 to 0.70)	775/162 102.7	0.48 (0.45 to 0.51)	506/162 373.6	0.31 (0.29 to 0.34)

b/tsDMARD, biologic or targeted synthetic disease-modifying antirheumatic drug; csDMARD, conventional synthetic DMARD; DVT, deep vein thrombosis;IR, incidence rate; PE, pulmonary embolism; VTE, venous thromboembolism.

Using the US Census population estimates on 1 July 2010, the age-standardised and sex-standardised IR of VTE was 0.86 (95% CI 0.70 to 1.03) per 100 patient-years in RA b/tsDMARD switchers, higher than that in the first b/tsDMARD users (0.60, 95% CI 0.52 to 0.68) and that in the csDMARD users (0.58, 95% CI 0.51 to 0.65). When we stratified by history of MI or stroke, the age-standardised and sex-standardised IR of VTE among those without a history of MI or stroke was 0.79 (0.62 to 0.97) in RA b/tsDMARD switchers, 0.53 (0.45 to 0.60) in the first b/tsDMARD users and 0.51 (0.43 to 0.58) in the csDMARD users. The standardised IR of VTE in first b/tsDMARD users was not different from that in csDMARD users in overall and the stratified analysis. Similar patterns were observed for DVT and PE (table 3). The majority of patients received TNF-α inhibitors as both an initial and subsequent (post-switch) therapy. When subgroups of b/tsDMARDs were explored, however, the age-standardised and sex-standardised VTE rates were comparable between TNF-α inhibitors and non-TNF-α biologics for both switchers and first users. Compared with TNF-α inhibitors and non-TNF-α biologics, the age-standardised and sex-standardised rate of VTE for tsDMARD was similar among first users but numerically lower among switchers, which might not be a reliable finding due to small numbers of tofacitinib users (the only tsDMARD evaluable in this study) (online supplemental table S3). The annual incidence for b/tsDMARD switchers, first b/tsDMARD users and csDMARD users was 0.75% (133/17 726), 0.57% (216/37 993) and 0.49% (457/92 509). Compared with csDMARD users, the NNH was 391 for b/tsDMARD switchers and 1342 for first b/tsDMARD users.

**Table 3:** Age and sex-standardised incidence rates and 95% CIs in b/tsDMARD switchers, first b/tsDMARD users and csDMARD users

	b/tsDMARD switchers	First b/tsDMARD users	csDMARD users
VTE	0.86 (0.70 to 1.03)	0.60 (0.52 to 0.68)	0.58 (0.51 to 0.65)
DVT	0.57 (0.44 to 0.70)	0.45 (0.38 to 0.52)	0.42 (0.36 to 0.48)
PE	0.48 (0.34 to 0.61)	0.27 (0.21 to 0.32)	0.26 (0.22 to 0.31)

Age and sex-standardised incidence rate was calculated by applying age and sex-specific incidence rates (ie, 18–44, 45–64, 65–74 and 75+ for men and women) to population age and sex distribution from US Census 2010 in a direct method,^47^ with 95% CIs estimated using normal approximation.^48^

b/tsDMARD, biologic or targeted synthetic disease-modifying antirheumatic drug; csDMARD, conventional synthetic DMARD; DVT, deep vein thrombosis; PE, pulmonary embolism; VTE, venous thromboembolism.

**Figure 3:**
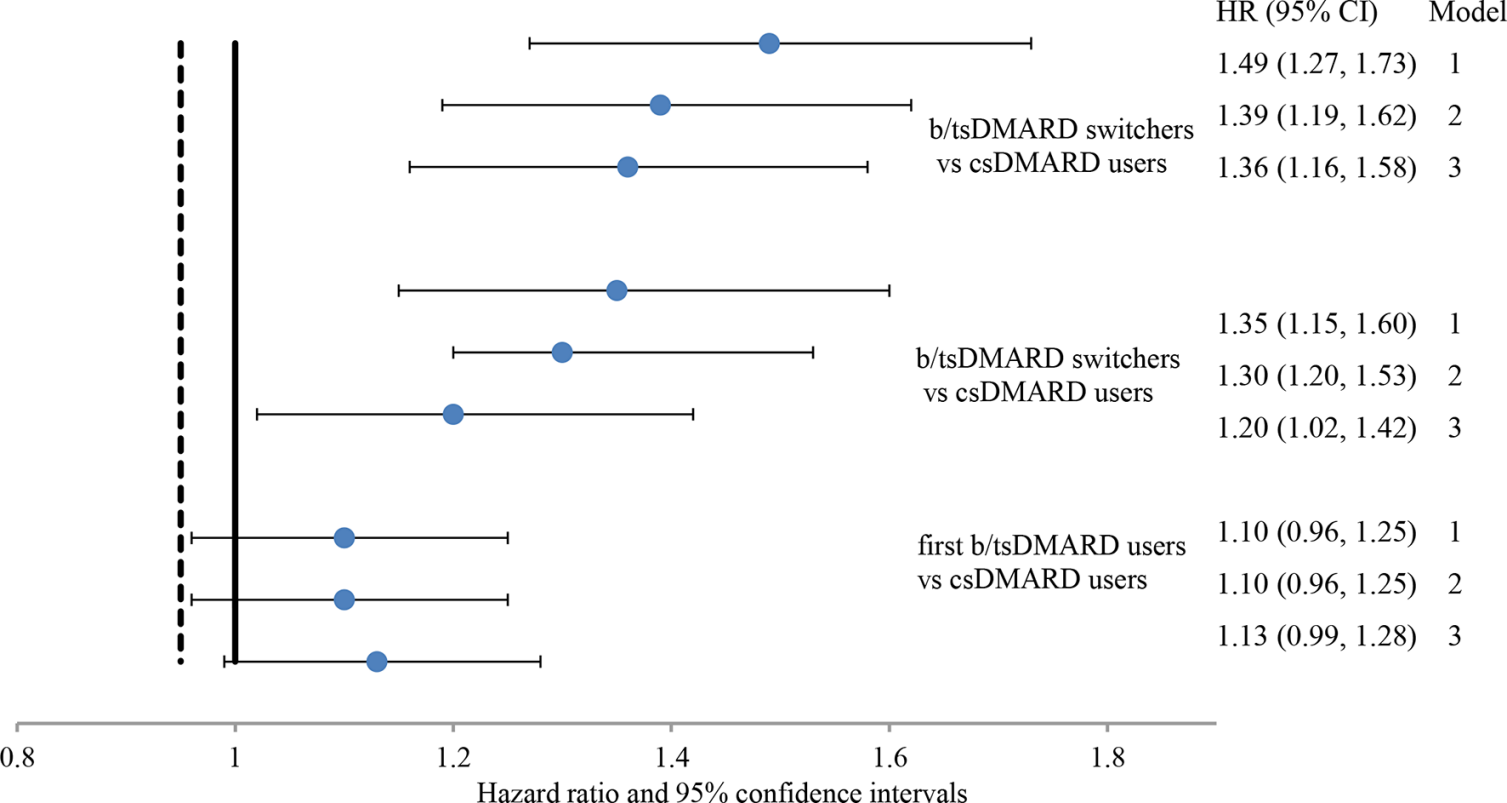
HRs and 95% CIs from multivariate Cox proportional-hazards model for venous thromboembolism (VTE) in patients with rheumatoid arthritis on biologic or non-biologic therapy. Note: model 1 adjusted for age, sex and race; model 2 adjusted for variables in model 1, plus history of VTE, and anticoagulant use at baseline; model 3 adjusted for variables in model 2, plus serious infections, hypertension, hospitalisation, chronic obstructive pulmonary disease, chronic kidney disease and myocardial infarction/stroke.

The adjusted HR (HR_adj_) and 95% CI for VTE from multivariate Cox regression are presented in figure 3. Model 1 is our primary model. When age, sex and race were adjusted (model 1), the risk of VTE among b/tsDMARD switchers was about 50% higher than that of csDMARD users and 35% higher than first b/tsDMARD users. After adjusting for additional variables including serious infections, hypertension, hospitalisations, COPD, CKD, MI or stroke, the HR_adj_ barely changed (from 1.39 in model 2 to 1.36 in model 3 in online supplemental table S4). The HRs were slightly attenuated with more risk factors adjusted from model 1 to model 2 to model 3, which suggested that the increased risk of VTE among b/tsDMARD switchers could be explained by differences in the medical histories. No significant difference in risk of VTE was identified between first b/tsDMARD users and csDMARDs users in any of these models. Similar patterns were observed for DVT and PE (online supplemental table S4). Other identified risk factors for VTE included history of VTE, anticoagulant use, serious infections, hypertension, hospitalisations, COPD, CKD and MI/stroke. The findings were consistent among those without a history of VTE, anticoagulant use and malignancy (online supplemental table S5).

In sensitivity analyses, the crude IR (95% CI) of VTE was 0.96 (0.78 to 1.16) per 100 PY for ‘second’ b/tsDMARD switchers and 0.87 (0.74 to 1.02) per 100 PY for ‘first’ b/tsDMARD switchers (online supplemental table S6). Compared with first b/tsDMARD users, controlling for age and sex and race, the HR (95% CI) for VTE was 1.48 (1.19 to 1.85) for ‘second’ b/tsDMARD switchers and 1.35 (1.15 to 1.60) for ‘first’ b/tsDMARD switchers (online supplemental table S7); in patients without a history of VTE, the HR remained significantly higher (second switchers HR 1.44, 95% CI 1.13 to 1.83; first switchers 1.29, 95% CI 1.07 to 1.56) (online supplemental table S8). The risk of DVT and PE followed similar patterns in all analyses.

## Discussion

In this analysis, we found that VTE risk was increased with advancing line of RA therapy, which may reflect line of therapy being a proxy for increasing disease severity and inadequate control of RA disease activity. With the increasing availability of b/tsDMARD options for RA, our finding that b/tsDMARD switching was associated with significantly increased VTE risk is particularly important for future observational studies of drug safety using administrative healthcare databases when standardised RA disease activity measures are unavailable. Specifically, we found an increased IR of VTE among patients with RA who switched from b/tsDMARDs to alternative b/tsDMARDs, when compared with either csDMARD users (HR_adj_ 1.36, 95% CI 1.16 to 1.58) or first b/tsDMARD users (HR_adj_ 1.20, 95% CI 1.02 to 1.42). The increased risk was more pronounced in the second-time b/tsDMARD switchers than in the first-time b/tsDMARD switchers. This study also confirmed several independent risk factors of VTE in RA patient population.

Our findings are consistent with the current literature on RA.^5 6^ Although the overall crude IR of VTE in this study (0.79, 95% CI 0.75 to 0.83) was somewhat higher than what was reported by Kim *et al* (0.61, 95% CI 0.54 to 0.69),^5^ this discrepancy is likely due to differences in study population and methodology. Kim *et al* studied a RA cohort from different data sources in an earlier calendar time period and excluded patients with claims for DVT or PE, or anticoagulants dispensing in the 12-month period before index date. Among similarly selected patients without anticoagulant use within 1 year before index date, the IR of VTE in our study (0.62, 95% CI 0.58 to 0.66 per 100 PY) was consistent with theirs.^5^

Unlike other reports, this study estimated the IR of VTE in the subgroup of patients with RA who switched from one b/tsDMARD to another. Switching from one b/tsDMARD to another b/tsDMARD may identify patients with insufficient response or intolerance to b/tsDMARD therapy in a real-world setting. Patients who are bDMARD users have been found to have more severe RA than patients treated with csDMARDs.^13 14^ In this study, despite on average being younger and with a similar pre-enrolment period, b/tsDMARD switchers had a higher proportion of serious infections, major knee and hip surgeries, and co-medication use indicating on average they had more severe RA. Since anti-TNF therapy appeared to be associated with reduced risk of VTE,^49^ the increased risk among b/tsDMARD switchers in this study could be due to continued inflammatory process^50^ (persistent disease severity) despite b/tsDMARD treatment and factors associated with treatment option, rather than treatments themselves. Although there was some attenuation in HR of VTE for b/tsDMARD switchers when additional risk factors reflecting comorbidities and disease severity were adjusted, even with adjustment of additional factors, b/tsDMARD switching remained associated with a significantly higher risk of VTE compared with first b/tsDMARD users or csDMARD users. As measures of RA disease activity or severity, such as Disease Activity Score or clinical disease activity index, were unavailable in claims data, switching b/tsDMARDs may be a proxy for higher disease severity or more poorly controlled RA.

We acknowledge that in a real-world setting, patients may switch RA treatments due to reasons such as insurance coverage or adverse events. However, the effect of such switching would be to underestimate, not overestimate, the differences in disease severity measures between the groups. Nevertheless, the increased risk in the second-time b/tsDMARD switchers, followed by the first-time b/tsDMARD switchers and the first b/tsDMARD users, demonstrated the utility of including switching b/tsDMARDs as a disease severity indicator.

In addition, this study confirmed several independent risk factors for VTE in patients with RA. Although risk factors such as increasing age, male sex and African-American race have been reported in general population, to the best of our knowledge, this is among the few US studies to report VTE risk factors that are relatively common in RA populations: history of VTE, history of anticoagulant use, serious infections, hypertension, hospitalisation, COPD, CKD and MI/stroke.

This study is subject to several limitations. First, the VTE outcome was identified based on ICD diagnosis on inpatient medical claims without further chart review. However, this method has been validated to have a positive predictive value ≥74%.^5 51^ Further, misclassification of the outcome is likely to be non-differential with respect to the treatment groups, thereby leading to bias towards the null. Second, we used b/tsDMARD switchers and number of switches (line of therapy) as our proxy for inadequate response to treatment and more severe RA. Misclassification of the number of previous cs/b/tsDMARDs used was possible due to availability of data (ie, left-censoring of events prior to enrolment in the health plan), which would have the conservative effect of biasing our results towards the null. It will be important to correctly classify patients’ biologic treatment history in future comparative safety analyses of VTE. Third, although several baseline medical conditions and medications were identified as independent risk factors in our analysis, RA disease activity or severity was unavailable in the database. Analysing RA registry or EHR data where disease activity and severity are available may help further understand the difference in risk of VTE among patients with different treatment experience. Further, this study identified inpatient VTE. Patients at low risk (eg, without CHF or severe liver impairment) for mortality may be treated as outpatients.^52^ However, outpatient VTE is less reliable and has not been used by comparative studies in RA.^5 6^ Although the incidence of VTE was underestimated, we used inpatient VTE to improve comparability between treatment groups. Fourth, stepwise regression was used in our analysis, the limitations of which have been described.^53^ Finally, this study focused on US patients treated with csDMARD or b/tsDMARD, and our study findings may not be generalisable to patients with RA in a different country.

In summary, this large US study provides incidence rates of VTE in patients with RA according to prior switching as a proxy for more severe disease in a real-world setting. Patients with RA with more extensive treatment experience, especially b/tsDAMRD switchers, have a higher risk of VTE compared with those treated with csDMARD and first b/tsDAMRD users. The heightened risk of VTE in b/tsDMARD switchers was somewhat attenuated when factors that reflect co-medications and comorbidities were controlled, yet remained significantly elevated, suggesting switching to an alternative b/tsDMARD may be a useful proxy for higher disease severity or poorly controlled RA in patients with other constellation of chronic conditions and risk factors. In addition to line of therapy, b/tsDMARD switching and the number of switches may be an important and independent risk factor to include moving forward in observational RA drug safety studies, particularly those using secondary data sources that lack standardised disease activity measures.
