# Supplementary material for: Incidence of inpatient venous thromboembolism in treated patients with rheumatoid arthritis and the association with switching biologic or targeted synthetic disease-modifying antirheumatic drugs (DMARDs) in the real-world setting
Source: RMD Open. 2019 Sep 23;5(2):e001013. doi: 10.1136/rmdopen-2019-001013 (PMC6803007; doi:10.1136/rmdopen-2019-001013)
Supplement: Supplementary data [file rmdopen-2019-001013supp001.pdf]

Supplemental materials for the study entitled "**Incidence of inpatient venous thromboembolism in treated rheumatoid arthritis patients and the association with switching biologic or targeted synthetic disease-modifying antirheumatic drugs (DMARDs) in the real-world setting**"

Supplemental data

Outcome, comorbidities, medical history and medications codes

Disease	Type	Codes
Rheumatoid arthritis	ICD-9	714.0
	ICD-10	M05.xx or M06.xx
VTE	ICD-9	451.1, 451.2, 451.9, 453.0, 453.1, 453.2, 453.3, 453.4, 453.8, 453.9, 451.81, 453.5, 453.9, 671.3, 671.4, 671.5, 415.1
	ICD-10	I80.1, I80.2, I80.3,I80.8, I80.9, I82.0, I82.1, I82.2, I82.3, I82.A, I82.B, I82.C, I82.4, I82.5, I82.6, I82.9, O22.3, O22.5, O22.9, O87.1, O87.3, O87.4, I26.xx
DVT	ICD-9	451.1, 451.2, 451.81, 451.9, 453.0, 453.1, 453.2, 453.3, 453.4, 453.5, 453.9
	ICD-10	I80.1, I80.2, I80.3, I80.9, I82.0, I82.1, I82.2, I82.3, I82.4, I82.5, I82.9
PE	ICD-9	415.1
	ICD-10	I26.xx
Inpatient diagnosis		Place of service = 21 hospital
alcohol abuse	ICD-9	291.xx, 303.xx, 980.xx, 760.71, 305.0x
	ICD-10	F10.xx, T51.xx, P04.3
drug abuse	ICD-9	291.xx, 303.xx, 980.xx, 304.xx, 305.xx
	ICD-10	F10.xx, F11.xx, F12.xx, F13.xx, F14.xx, F15.xx, F16.xx, F17.xx, F18.xx, F19.xx, T51.xx
smoking history	ICD-9	305.1
	ICD-10	F17.xx, Z72.0
Obesity	ICD-9	278.00, 278.01, V85.3x, V85.4x
	ICD-10	E66.0x, E66.1, E66.2x, E66.8, E66.9, Z68.3x, Z68.4x
hypertension	ICD-9	401.xx, 402.xx, 403.xx, 404.xx
	ICD-10	I10.xx, I11.xx, I12.xx, I13.xx
diabetes mellitus	ICD-9	250.xx
	ICD-10	E10.xx, E11.xx
hyperlipidemia	ICD-9	272.0, 272.1, 272.2, 272.3, 272.4
	ICD-10	E78.0x, E78.1, E78.2, E78.3, E78.4, E78.5
congestive heart failure	ICD-9	428.xx
	ICD-10	I50.xx
myocardial infarction (disease history)	ICD-9	410.xx, 412.xx
	ICD-10	I21.xx, I22.xx, I23.xx
Stroke (disease history)	ICD-9	430.xx, 431.xx, 432.xx, 433.xx, 434.xx, 435.xx, 436.xx, 437.xx, 438.xx
	ICD-10	I60.xx, I61.xx, I62.xx, I63.xx, I65.xx, I66.xx, I67.xx, I68.xx, I69.xx, Z86.73
death	ICD-9	798.1, 798.2, 798.9
	ICD-10	R99
**malignancy**	ICD-9	140.xx-165.xx, 170.xx-173.xx, 174.xx-176.xx, 179.xx-194.xx, 200.xx-208.xx
	ICD-10	C00.xx-C44.xx, C45.xx-C75.xx, C81.xx-C95.xx
**serious**	ICD-9	Agency for Healthcare Research and Quality (AHRQ) QI™ ICD-9-CM Specification

Liang H, *et al. RMD Open* 2019; 5:e001013. doi: 10.1136/rmdopen-2019-001013

**infections** (diagnosis on inpatient claims)		version 6.0 Patient Safety Indicators (PSI) Appendix F Infection Diagnosis Codes. Available from URL: https://www.qualityindicators.ahrq.gov/Downloads/Modules/PSI/V60-ICD09/TechSpecs/PSI_Appendix_F.pdf Accessed 1 July 2018
	ICD-10	Agency for Healthcare Research and Quality (AHRQ) QI[TM] ICD-10-CM Specification v2018 Patient Safety Indicators (PSI) Appendix F Infection Diagnosis Codes. Available from URL: https://www.qualityindicators.ahrq.gov/Downloads/Modules/PSI/V2018/TechSpecs/PSI_Appendix_F.pdf Accessed 1 July 2018
HIV infection	ICD-9	042, V08, 079.53, 795.71
	ICD-10	B20, Z21, O98.7xx
viral hepatitis	ICD-9	070.xx
	ICD-10	B15.xx, B16.xx, B17.xx, B18.xx, B19.xx
diverticular disease	ICD-9	562.xx
	ICD-10	K57.xx
gastroduodenal ulcer	ICD-9	531.xx, 532.xx, 533.xx
	ICD-10	K25.xx, K26.xx, K27.xx
COPD	ICD-9	490.xx, 491.xx, 492.xx, 494.xx, 496.xx
	ICD-10	J40.xx, J41.xx, J42.xx, J43.xx, J44.xx, J47.xx
asthma	ICD-9	493.xx
	ICD-10	J45.xx
inflammatory bowel disease	ICD-9	556.xx, 555.xx, 558.9
	ICD-10	K51.xx, K50.xx, K52.3, K52.89, K52.9
depression	ICD-9	296.2x, 296.3x, 293.83, 300.4x
	ICD-10	F34.1, F32.xx, F33.xx, F06.30, F06.31, F06.32
Atrial fibrillation/flutter	ICD-9	427.31, 427.32
	ICD-10	I48.xx
Hip fracture	ICD-9	733.1x, 733.81, 733.82, 800.xx-829.xx, 905.0-905.5, V54.xx
	ICD-10	S02.xx, S12.xx, S22.xx, S32.xx, S42.xx, S49.xx, S52.xx, S59.xx, S62.xx, S72.xx, S79.xx, S82.xx, S89.xx, S92.xx, M84.x
	CPT	22310-21495, 21800-21825, 23500-23515, 23570-23630, 23500-23515, 24500-24582, 24582, 24650-24685, 25500-25652, 26600-26615, 26720-26765, 27193-27248, 27267-27269, 27500-27540, 2750-27828, 28400-28531, 29847-29856
Hip or knee surgery	CPT	27299, S2325, 27125,27130,27299, S2118,27130, 27132, 27134, 27137, 27138,27120,27122,27125, 27445, 27447, 27486, 27487,27446, 29914, 29915, 29916, 28446, 27412, 29866,29867, 27415,27416
Smoking	CPT/HCPCS	"99406" "99407" "G0436" "G0437"
Oral contraceptive use	ICD-9	V25.xx
	ICD-10	Z30.xx
	ICD-9-procedure	65.6x, 66.2x, 66.3x, 66.5x, 66.69, 66.97
	ICD-10-procedure	0U57xxx, 0UL7xxx, 0UT7xxx
	CPT	58300, 58301
	HCPCS	J1050, J7300, J7301, J7302, J7303, J7304, J7307, S4993, AA261, A4266, A4267, A4268
Drug abuse	CPT/HCPCS	"99408" "99409" "G0396" "G0397" "H0001" "H0002" "H0005" "H0006" "H0007" "H0008" "H0009" "H0010" "H0011" "H0012" "H0013" "H0014" "H0015" "H0016" "H0018" "H0019" "H0020" "H0021" "H0022" "H0047" "H0048" "H0049" "H0050" "H2034""H2035" "H2036" "T1006" "T1007"
Corticosteroi	ICD-9-	V58.65

ds	CM	
	ICD-10-CM	Z79.51, Z79.52
NSAIDs	ICD-9-CM	V58.64
	ICD-10-CM	Z79.1
Antithrombotics	ICD-9-CM	V58.61, V58.63
	ICD-10-CM	Z79.02, Z79.01
Therapeutic Anticoagulants	ICD-9-CM	Z58.83 (therapeutic drug monitoring) in combination with V58.61 (anticoagulant use)
	ICD-10-CM	Z5181 (therapeutic drug monitoring) in combination with Z7901 (anticoagulant use)
Corticosteroids	ICD-9 procedure	99.23
	ICD-10-PCS	3E00X3Z, 3E0133Z, 3E0233Z, 3E0303Z, 3E0333Z, 3E0403Z, 3E0433Z, 3E0503Z, 3E0533Z, 3E0603Z, 3E0633Z, 3E0933Z, 3E0973Z, 3E09X3Z, 3E0B33Z, 3E0B73Z, 3E0BX3Z, 3E0C33Z, 3E0C73Z, 3E0CX3Z, 3E0D33Z, 3E0D73Z, 3E0proc3Z, 3E0E33Z, 3E0E73Z, 3E0E83Z, 3E0F33Z, 3E0F73Z, 3E0F83Z, 3E0G33Z, 3E0G73Z, 3E0G83Z, 3E0H33Z, 3E0H73Z, 3E0H83Z, 3E0J33Z, 3E0J73Z, 3E0J83Z, 3E0K33Z, 3E0K73Z, 3E0K83Z, 3E0L33Z, 3E0M33Z, 3E0N33Z, 3E0N73Z, 3E0N83Z, 3E0P33Z, 3E0P73Z, 3E0P83Z, 3E0Q03Z, 3E0Q33Z, 3E0R33Z,3E0S33Z, 3E0T33Z, 3E0U33Z, 3E0V33Z, 3E0W33Z, 3E0X33Z, 3E0Y33Z
Anticoagulants	ICD-9 procedure	99.19
	ICD-10-PCS	XW03392, XW04392
Antiplatelet agents	ICD-9 procedure	99.20
	ICD-10-PCS	3E033PZ, 3E043PZ, 3E053PZ, 3E063PZ, 3E083PZ
Thrombolytic agents	ICD-9 procedure	99.10
	ICD-10-PCS	3E03017, 3E030PZ, 3E03317, 3E04017, 3E040PZ, 3E04317, 3E05017, 3E050PZ, 3E05317, 3E06017, 3E060PZ, 3E06317, 3E08017, 3E080PZ, 3E08317
Corticosteroids	AHFSCLSS	"481008"
NSAIDs	AHFSCLSS	"280804" "28080408" "28080424" "28080492"
Cox-2 inhibitors	AHFSCLSS	"28080408"
Salicylates	AHFSCLSS	"28080424"
Other NSAIDs	AHFSCLSS	"28080492"
Antithrombotic agents	AHFSCLSS	"201204" "201218" "201220"
Anticoagulants	AHFSCLSS	"201204"
Antiplatelet agents	AHFSCLSS	"201218"

Thrombolytic agents	AHFSCLSS	"201220"
Corticosteroids	CPT/HCPCS	"J1094" "J3300" "J3300" "J3302" "J3303" "J7622" "J7626" "J7627" "J7633" "J7634" "J7637" "J7638" "J7641" "J7683" "J7684" "J8540" "K0512" "K0513" "K0527" "K0528" "Q0137" "Q0138" "S0173" "S1090" "4135F" "4140F"
NSAIDs	CPT/HCPCS	"G8895" "G9201" "G9435" "G9793" "4044F" "4070F" "C9279" "J1130" "J1741" "J1885"
Salicylates	CPT/HCPCS	"G8895" "G9201" "G9435" "G9793" "4044F" "4070F"
Other NSAIDs	CPT/HCPCS	"C9279" "J1130" "J1741" "J1885"
Antiplatelet agents	CPT/HCPCS	"G8579" "G9438" "G8222" "G8224" "G9609" "4011F" "4073F" "C9460" "J0130" "J1327" "J3246" "J3364" "J3365" "C9109" "J3245" "C9460" "G8521"
Thrombolytic agents	CPT/HCPCS	"G8696" "86590" "J0350" "J2993" "J2994" "J2995" "J2996" "J2997" "J3101" "C9002" "C9005" "J3100"
Antithrombotics	CPT/HCPCS	
Anticoagulants	CPT/HCPCS	category_dtl_code_desc in ("DME AND SUPPLIES" "MEDICATIONS" "NOT ASSIGNED" "OTHER THERAPEUTIC PROCEDURES") and "G8799" "G8967" "4075F" "A4800" "A4801" "C9107" "C9111" "C9121" "E1520" "J0583" "J0883" "J1642" "J1644" "J1645" "J1650" "J1652" "J1655" "J1945" "J7197" "Q2021" "Y5273" "99363" "99364" "93793" "S9336" "S9372"

ICD-9-CM, ICD-10-CM, ICD-9 procedure, ICD-10 procedure, AHFSCLSS codes and CPT codes were used to corticosteroids, NSAIDs and anticoagulants

Biologic or targeted synthetic disease-modifying antirheumatic drugs (b/tsDMARD), conventional DMARD (cDMARD) in patients with rheumatoid arthritis

Treatment	Mechanism of action	Generic name
bDMARD	TNF-α inhibitors	etanercept, adalimumab, certolizumab, golimumab, infliximab
	non-TNF biologics	abatacept, rituximab, anakinra, tocilizumab, sarilumab
tsDMARDs	JAK-inhibitors	tofacitinib and baricitinib (Baricitinib was neither approved nor available in the data during the study window)
csDMARD	Other immunosuppressant	methotrexate, hydroxychloroquine, sulfasalazine, and leflunomide

Compared with intent-to-treat analysis approach, on-treatment approach is more conservative for the reasons below

$$\frac{N}{D}-\frac{N+X}{D+Y}=\frac{NY-DX}{D(D+Y)}=\frac{\frac{N}{D}Y-X}{D+Y}$$

N: Total number of VTE events

D: Total number of patient-years

$\frac{N}{D}$: Incidence rate using treatment emergent AE approach

X: Observed additional number of events using ever-exposure model

Y: Additional number of patient-years using ever-exposure model

$\frac{N+X}{D+Y}$: Incidence rate using ever-exposure model

$\frac{N}{D}Y$: Expected additional events assuming the incidence rate is consistent across time

Liang H, *et al. RMD Open* 2019; 5:e001013. doi: 10.1136/rmdopen-2019-001013

Conclusion: If the incidence rate of VTE decreases after drug discontinuation (a likely scenario if the drug is associated with VTE), the intention to treat model will underestimate the incidence rate while on-treatment approach will not (as $\frac{N}{D}Y - X$ will always be greater than 0).

Liang H, *et al. RMD Open* 2019; 5:e001013. doi: 10.1136/rmdopen-2019-001013

Supplemental Table S1a - Demographics and baseline characteristics of patients with rheumatoid arthritis on DMARD therapy

	b/tsDMARD switchers (1)	First b/tsDMARD users (2)	csDMARD users (3)	Std. diff. 1 vs 3	Std. diff. 2 vs 3	Std. diff. 1 vs 2
N	17,726 (100.0)	37,993 (100.0)	92,509 (100.0)	-	-	-
Year of cohort entry						
2007-2009	3,910 (22.1)	15,776 (41.5)	33,432 (36.1)	**-0.312**	**0.184**	**-0.502**
2010-2012	4,672 (26.4)	8,141 (21.4)	20,649 (22.3)	0.093	-0.022	**0.115**
2013-2015	5,742 (32.4)	8,171 (21.5)	21,676 (23.4)	**0.202**	-0.046	**0.248**
2016-2017	3,402 (19.2)	5,905 (15.5)	16,752 (18.1)	0.028	-0.070	0.098
Geographic region						
Northeast	1,359 (7.7)	3,103 (8.2)	8,261 (8.9)	-0.044	-0.025	-0.018
Midwest	4,127 (23.3)	8,641 (22.7)	19,533 (21.1)	0.053	0.039	0.014
South	8,571 (48.4)	17,733 (46.7)	41,964 (45.4)	0.060	0.026	0.034
West	3,623 (20.4)	8,396 (22.1)	22,477 (24.3)	-0.094	-0.052	-0.042
Unknown	46 (0.3)	120 (0.3)	274 (0.3)	0.000	0.000	0.000
Alcohol abuse	266 (1.5)	462 (1.2)	1181 (1.3)	0.017	-0.009	0.026
**Medical history**						
Obesity	2302 (13)	3723 (9.8)	9685 (10.5)	0.078	-0.023	**0.101**
Viral hepatitis	196 (1.1)	414 (1.1)	989 (1.1)	0	0	0
Diverticular disease	872 (4.9)	1518 (4.0)	4994 (5.4)	-0.023	-0.066	0.044
Gastro duodenal ulcer	347 (2.0)	612 (1.6)	1661 (1.8)	0.015	-0.015	0.030
HIV infection	16 (0.09)	37 (0.10)	110 (0.12)	-0.009	-0.006	-0.003
Inpatient hospital stays (days)						
≥7 days	863 (4.9)	1569 (4.1)	5295 (5.7)	-0.036	-0.074	0.039
1-<7 years	1843 (10.4)	3522 (9.3)	9213 (10.0)	0.013	-0.024	0.037
0 day	15020 (84.7)	32902 (86.6)	78001 (84.3)	0.011	0.065	-0.054

Abbreviation: DMARD= disease modifying antirheumatic drugs; b/tsDMARD= biologic or targeted synthetic DMARD; csDMARD=conventional synthetic DMARD; Std. diff.=standardized difference; HIV= human immunodeficiency virus.
Note: Data are presented as n (%), unless otherwise specified. A standardized difference with an absolute value of greater than 0.10 indicates statistical significance for groups under comparison.

Liang H, *et al. RMD Open* 2019; 5:e001013. doi: 10.1136/rmdopen-2019-001013

Supplemental Table S1b –Drugs used at cohort entry in patients with rheumatoid arthritis on DMARD therapy n (%)

Index drug	b/tsDMARD switchers (n=17,726)	First b/tsDMARD users (n=37,993)	Index drug	csDMARD users (n=92,509)
ABATACEPT	2866(16.2)	2007(5.3)	APREMILAST	76(0.1)
ADALIMUMAB	4279(24.1)	11123(29.3)	AURANOFIN	68(0.1)
ANAKINRA	41(0.2)	103(0.3)	AZATHIOPRINE	1769(1.9)
CERTOLIZUMAB	1737(9.8)	1243(3.3)	CYCLOPHOSPHAMIDE	132(0.1)
ETANERCEPT	2664(15.0)	13763(36.2)	CYCLOSPORINE	3287(3.6)
GOLIMUMAB	1647(9.3)	1255(3.3)	GOLD-THIOMALATE	56(0.1)
INFLIXIMAB	1596(9)	6225(16.4)	HYDROXYCHLOROQUINE	31888(34.5)
INFLIXIMAB-DYYB	23(0.1)	8(0.0)	LEFLUNOMIDE	4516(4.9)
RITUXIMAB	1046(5.9)	1189(3.1)	METHOTREXATE	42489(45.9)
SARILUMAB	9(0.1)	4(0.0)	MINOCYCLINE	2292(2.5)
TOCILIZUMAB	971(5.5)	393(1.0)	MYCOPHENOLATE	680(0.7)
TOFACITINIB	847(4.8)	680(1.8)	PENICILLAMINE	27(0.0)
			SULFASALAZINE	5229(5.7)

Liang H, *et al. RMD Open* 2019; 5:e001013. doi: 10.1136/rmdopen-2019-001013

Supplemental Table S2 - Incidence rate of VTE, DVT and PE per 100 patient-years by treatments, demographics and baseline characteristics in patients with rheumatoid arthritis

		Inpatient VTE		Inpatient DVT		Inpatient PE	
	N	Cases/PY	IR (95% CI)	Cases/PY	IR (95% CI)	Cases/PY	IR (95% CI)
All	148,228	1295/164476.8	0.79 (0.75-0.83)	936/164742.2	0.57 (0.53-0.61)	596/165047.5	0.36 (0.33-0.39)
**Medical history**							
Hypertension							
Yes	70,963	917/74064.2	1.24 (1.16-1.32)	660/74248.5	0.89 (0.82-0.96)	414/74470.8	0.56 (0.50-0.61)
No	77,265	378/90412.6	0.42 (0.38-0.46)	276/90493.7	0.30 (0.27-0.34)	182/90576.7	0.20 (0.17-0.23)
Myocardial infarction or stroke							
Yes	12,777	272/11847.5	2.30 (2.03-2.59)	207/11891.9	1.74 (1.51-1.99)	108/11971.7	0.90 (0.74-1.09)
No	135,451	1023/152629.3	0.67 (0.63-0.71)	729/152850.3	0.48 (0.44-0.51)	488/153075.8	0.32 (0.29-0.35)
Chronic kidney disease							
Yes	19,104	324/17483.2	1.85 (1.66-2.07)	255/17539.2	1.45 (1.28-1.64)	130/17610.2	0.74 (0.62-0.88)
No	129,124	971/146993.6	0.66 (0.62-0.70)	681/147203	0.46 (0.43-0.5)	466/147437.3	0.32 (0.29-0.35)
Cancer							
Yes	9067	115/8797.4	1.31 (1.08-1.57)	77/8821.4	0.87 (0.69-1.09)	54/8852.9	0.61 (0.46-0.8)
No	139,161	1180/155679.4	0.76 (0.72-0.8)	859/155920.8	0.55 (0.51-0.59)	596/165047.5	0.36 (0.33-0.39)
Serious infections							
Yes	21,304	441/20370.4	2.16 (1.97-2.38)	313/20463.2	1.53 (1.36-1.71)	186/20558.6	0.90 (0.78-1.04)
No	126,924	854/144106.4	0.59 (0.55-0.63)	623/144279.1	0.43 (0.40-0.47)	410/144488.9	0.28 (0.26-0.31)
COPD							
Yes	17,839	321/16441.2	1.95 (1.74-2.18)	215/16514.3	1.30 (1.13-1.49)	152/16540.1	0.92 (0.78-1.08)
No	130,389	974/148035.6	0.66 (0.62-0.70)	721/148227.9	0.49 (0.45-0.52)	444/148507.4	0.30 (0.27-0.33)
**Medication use history**							
NSAID							
Yes	109,053	916/118979.9	0.77 (0.72-0.82)	669/119166.6	0.56 (0.52-0.61)	419/119412.4	0.35 (0.32-0.39)
No	39,175	379/45497	0.83 (0.75-0.92)	267/45575.6	0.59 (0.52-0.66)	177/45635.1	0.39 (0.33-0.45)
Cox-2 inhibitors							
Yes	30,100	293/36260.7	0.81 (0.72-0.91)	229/36311.6	0.63 (0.55-0.72)	117/36443.8	0.32 (0.27-0.38)
No	118,128	1002/128216.1	0.78 (0.73-0.83)	707/128430.6	0.55 (0.51-0.59)	479/128603.7	0.37 (0.34-0.41)
Corticosteroids							
Yes	38,464	462/41201.3	1.12 (1.02-1.23)	330/41277.3	0.80 (0.72-0.89)	212/41398.7	0.51 (0.45-0.59)
No	109,764	833/123275.5	0.68 (0.63-0.72)	606/123464.9	0.49 (0.45-0.53)	384/123648.8	0.31 (0.28-0.34)

Abbreviation: VTE= venous thromboembolism; DVT=deep vein thrombosis; PE=pulmonary embolism; COPD= Chronic obstructive pulmonary disease; IBD=inflammatory bowel disease; NSAID= Nonsteroidal anti-inflammatory drugs.

Liang H, *et al. RMD Open* 2019; 5:e001013. doi: 10.1136/rmdopen-2019-001013

Supplemental Table S3 – Incidence rate of VTE for subgroups of b/tsDMARD users

	N	Female (%)	Mean age ± SD	Cases/PY	Crude IR (95% CI)	Age and sex standardized rate (95% CI)
Treatment						
b/tsDMARD switchers	17,726	78.7	54.6 ± 12.8	229/26715	0.86 (0.75-0.98)	0.86 (0.70-1.03)
TNF-α inhibitors	11,923	77.2	53.2 ± 12.6	160/18929.5	0.85 (0.72-0.99)	0.93 (0.71, 1.14)
Non-TNF-α biologics	4933	81.7	57.3 ± 12.8	65/6879.0	0.94 (0.73-1.20)	0.85 (0.51, 1.20)
Tofacitinib*	847	82.2	57.8 ± 12.5	4/898.8	0.45 (0.12-1.14)	0.17 (0.00, 0.33)
Biosimilars	23	82.6	70.0 ± 12.0	0/7.7	NA	NA
First b/tsDMARD users	37,993	75.0	54.7 ± 13.4	364/54138.1	0.67 (0.61-0.75)	0.60 (0.52-0.67)
TNF-α inhibitors	33,609	74.3	54.0 ± 13.2	319/50306.5	0.63 (0.57-0.71)	0.57 (0.49-0.65)
Non-TNF-α biologics	3696	80.2	59.8 ± 13.9	39/3284.5	1.19 (0.84-1.62)	0.89 (0.53-1.26)
Tofacitinib	680	80.6	60.4 ± 12.9	6/546.1	1.10 (0.4-2.39)	0.82 (0.03-1.60)
Biosimilars	8	87.5	63.3 ± 7.7	0/1.1	NA	NA

Abbreviation: b/tsDMARD= biologic or targeted synthetic disease modifying antirheumatic drugs (DMARD); csDMARD=conventional synthetic DMARD; VTE= venous thromboembolism; DVT=deep vein thrombosis; PE=pulmonary embolism.
Age and Sex-standardized incidence rate was calculated by applying age- and sex-specific incidence rates (i.e. 18-44, 45-64, 65-74, and 75+ for men and women) to population age and sex distribution from US Census 2010 in a direct method[45], with 95% confidence intervals estimated using normal approximation
*Baricitinib was not available during the study window.

Liang H, *et al. RMD Open* 2019; 5:e001013. doi: 10.1136/rmdopen-2019-001013

Supplemental Table S4 – Hazard ratios and 95% confidence intervals from multivariate Cox proportional hazard model for venous thromboembolism in patients with rheumatoid arthritis on DMARD therapy to be cont'd

Independent variables	**Model 1**	**Model 2**	**Model 3**
**Venous thromboembolism**			
**b/tsDMARD switchers vs csDMARDs users**	**1.49 (1.27, 1.73)**	**1.39 (1.19, 1.62)**	**1.36 (1.16, 1.58)**
**b/tsDMARD switchers vs first users**	**1.35 (1.15, 1.60)**	**1.30 (1.20, 1.53)**	**1.20 (1.02, 1.42)**
**First b/tsDMARD users vs csDMARDs users**	**1.10 (0.96, 1.25)**	**1.10 (0.96, 1.25)**	**1.13 (0.99, 1.28)**
Age 45-64 vs 18-44	1.59 (1.28, 1.99)	1.43 (1.14, 1.78)	1.18 (0.94, 1.48)
Age 65-74 vs 18-44	3.39 (2.70, 4.26)	2.69 (2.14, 3.39)	1.89 (1.49, 2.40)
Age 75+ vs 18-44	4.56 (3.61, 5.76)	3.24 (2.55, 4.10)	2.10 (1.64, 2.69)
Sex (Male vs Female)	1.22 (1.08, 1.38)	1.20 (1.06, 1.35)	1.17 (1.04, 1.32)
Black vs White	1.19 (0.99, 1.43)	1.16 (0.97, 1.39)	1.02 (0.85, 1.23)
Asian vs White	0.65 (0.42, 1.01)	0.72 (0.46, 1.13)	0.74 (0.48, 1.15)
Hispanic vs White	0.73 (0.59, 0.90)	0.80 (0.65, 0.98)	0.80 (0.65, 0.99)
Unknown race vs White	0.79 (0.63, 0.98)	0.81 (0.65, 1.01)	0.84 (0.67, 1.04)
VTE history		5.61 (4.77, 6.60)	4.07 (3.46, 4.78)
Anticoagulants use		1.83 (1.59, 2.11)	1.53 (1.32, 1.78)
Serious infections			1.44 (1.25, 1.66)
Hypertension			1.54 (1.34, 1.76)
Hospitalization 7+ days vs 0 day			1.84 (1.54, 2.20)
Hospitalization 1-6 days vs 0 day			1.32 (1.12, 1.55)
Chronic obstructive pulmonary disease			1.28 (1.12, 1.48)
Chronic kidney disease			1.28 (1.12, 1.47)
Myocardial infarction or stroke			1.26 (1.09, 1.46)
**Deep vein thrombosis**			
b/tsDMARD switchers vs first users	1.35 (1.11, 1.64)	1.29 (1.06, 1.57)	1.20 (0.99, 1.46)
b/tsDMARD switchers vs csDMARDs users	1.48 (1.24, 1.78)	1.43 (1.19, 1.71)	1.36 (1.13, 1.63)
First b/tsDMARD users vs csDMARDs users	1.10 (0.95, 1.29)	1.10 (0.95, 1.29)	1.13 (0.97, 1.32)
Age 45-64 vs 18-44	1.36 (1.06, 1.75)	1.24 (0.97, 1.59)	1.01 (0.79, 1.31)
Age 65-74 vs 18-44	2.94 (2.27, 3.8)	2.41 (1.86, 3.11)	1.63 (1.25, 2.14)
Age 75+ vs 18-44	4.29 (3.30, 5.58)	3.15 (2.41, 4.10)	1.97 (1.49, 2.60)
Sex (Male vs Female)	1.20 (1.04, 1.38)	1.17 (1.01, 1.35)	1.15 (0.99, 1.32)
Black vs White	1.13 (0.91, 1.40)	1.11 (0.89, 1.37)	0.97 (0.78, 1.20)
Asian vs White	0.45 (0.24, 0.83)	0.48 (0.26, 0.90)	0.50 (0.27, 0.93)
Hispanic vs White	0.76 (0.60, 0.96)	0.83 (0.65, 1.05)	0.82 (0.65, 1.04)
Unknown race vs White	0.81 (0.63, 1.05)	0.82 (0.64, 1.07)	0.86 (0.66, 1.11)
VTE history		5.80 (4.80, 7.01)	4.13 (3.41, 4.99)
Anticoagulants use		1.92 (1.60, 2.30)	1.47 (1.22, 1.75)

Serious infections	1.38 (1.17, 1.63)
Hypertension	1.51 (1.28, 1.77)
Hospitalization 7+ days vs 0 day	1.87 (1.52, 2.31)
Hospitalization 1-6 days vs 0 day	1.29 (1.07, 1.57)
Chronic obstructive pulmonary disease	1.12 (0.95, 1.33)
Chronic kidney disease	1.48 (1.26, 1.73)
Myocardial infarction or stroke	1.38 (1.16, 1.64)

Abbreviation: DMARD= disease modifying antirheumatic drugs; b/tsDMARD= biologic or targeted synthetic DMARD; csDMARD=conventional synthetic DMARD; Cases=number of patients with an outcome event; N=size of the patient population; HR=hazard ratio; CI=confidence interval; VTE= venous thromboembolism; DVT=deep vein thrombosis; PE=pulmonary embolism.

Supplemental Table S4 – Hazard ratios and 95% confidence intervals from multivariate Cox proportional hazard model for venous thromboembolism in patients with rheumatoid arthritis on DMARD therapy Cont'd

Independent variables	Model 1	Model 2	Model 3
**Pulmonary embolism**			
b/tsDMARD switchers vs first users	1.48 (1.17, 1.88)	1.43 (1.13, 1.81)	1.33 (1.05, 1.69)
b/tsDMARD switchers vs csDMARDs users	1.59 (1.27, 1.98)	1.53 (1.23, 1.91)	1.47 (1.18, 1.83)
First b/tsDMARD users vs csDMARDs users	1.07 (0.88, 1.30)	1.07 (0.88, 1.30)	1.10 (0.91, 1.34)
Age 45-64 vs 18-44	1.75 (1.26, 2.43)	1.62 (1.17, 2.26)	1.33 (0.95, 1.87)
Age 65-74 vs 18-44	3.47 (2.47, 4.87)	2.91 (2.07, 4.08)	2.04 (1.43, 2.90)
Age 75+ vs 18-44	4.25 (2.99, 6.05)	3.25 (2.28, 4.64)	2.13 (1.47, 3.08)
Sex (Male vs Female)	1.22 (1.02, 1.45)	1.19 (1.00, 1.42)	1.17 (0.98, 1.40)
Black vs White	1.14 (0.87, 1.49)	1.12 (0.85, 1.46)	1.00 (0.76, 1.31)
Asian vs White	0.85 (0.48, 1.51)	0.92 (0.52, 1.63)	0.97 (0.55, 1.73)
Hispanic vs White	0.61 (0.44, 0.85)	0.65 (0.47, 0.91)	0.67 (0.48, 0.93)
Unknown race vs White	0.86 (0.63, 1.17)	0.88 (0.64, 1.20)	0.90 (0.66, 1.23)
VTE history		4.82 (3.76, 6.18)	3.69 (2.87, 4.73)
Anticoagulants use		1.89 (1.50, 2.38)	1.54 (1.22, 1.94)
Serious infections			1.47 (1.19, 1.81)
Hypertension			1.64 (1.35, 2.00)
Chronic obstructive pulmonary disease			1.55 (1.26, 1.89)
Hospitalization 7+ days vs 0 day			1.48 (1.12, 1.95)
Hospitalization 1-6 days vs 0 day			1.18 (0.93, 1.51)
Cox-2 inhibitors			0.78 (0.63, 0.95)

Abbreviation: DMARD= disease modifying antirheumatic drugs; b/tsDMARD= biologic or targeted synthetic DMARD; csDMARD=conventional synthetic DMARD; Cases=number of patients with an outcome event; N=size of the patient population; HR=hazard ratio; CI=confidence interval; VTE= venous thromboembolism; DVT=deep vein thrombosis; PE=pulmonary embolism.

Liang H, *et al. RMD Open* 2019; 5:e001013. doi: 10.1136/rmdopen-2019-001013

Supplemental Table S5 – Hazard ratios and 95% confidence intervals from multivariate Cox proportional hazard model for venous thromboembolism in patients with rheumatoid arthritis on DMARD therapy without a history of VTE, anticoagulant use, malignancy and HIV infection

Independent variables	VTE	DVT	PE
b/tsDMARD switchers vs first users	1.25 (1.01, 1.55)	1.26 (0.98, 1.63)	1.42 (1.06, 1.92)
b/tsDMARD switchers vs csDMARDs users	1.53 (1.24, 1.88)	1.51 (1.18, 1.93)	1.66 (1.25, 2.2)
First b/tsDMARD users vs csDMARDs users	1.22 (1.03, 1.44)	1.2 (0.98, 1.46)	1.16 (0.91, 1.48)
Age 45-64 vs 18-44	1.76 (1.33, 2.33)	1.56 (1.13, 2.16)	1.64 (1.12, 2.4)
Age 65-74 vs 18-44	3.71 (2.77, 4.96)	3.3 (2.35, 4.63)	3.44 (2.31, 5.14)
Age 75+ vs 18-44	5.27 (3.89, 7.15)	5.52 (3.9, 7.8)	4.46 (2.92, 6.8)
Sex (Male vs Female)	1.22 (1.04, 1.43)	1.21 (1, 1.46)	1.15 (0.92, 1.45)
Black vs White	1.11 (0.87, 1.42)	1 (0.74, 1.35)	1.17 (0.83, 1.64)
Asian vs White	0.65 (0.38, 1.13)	0.48 (0.23, 1.02)	0.71 (0.33, 1.5)
Hispanic vs White	0.69 (0.53, 0.91)	0.72 (0.53, 0.99)	0.55 (0.36, 0.84)
Unknown race vs White	0.74 (0.55, 1)	0.72 (0.51, 1.03)	0.86 (0.58, 1.27)

Abbreviation: DMARD= disease modifying antirheumatic drugs; b/tsDMARD= biologic or targeted synthetic DMARD; csDMARD=conventional synthetic DMARD; Cases=number of patients with an outcome event; N=size of the patient population; HR=hazard ratio; CI=confidence interval; VTE= venous thromboembolism; DVT=deep vein thrombosis; PE=pulmonary embolism.

Liang H, *et al. RMD Open* 2019; 5:e001013. doi: 10.1136/rmdopen-2019-001013

Supplemental Table S6 - Incidence rate of VTE, DVT and PE per 100 patient-years by treatments in patients with rheumatoid arthritis

		Inpatient VTE		Inpatient DVT		Inpatient PE	
	N	Cases/PY	IR (95% CI)	Cases/PY	IR (95% CI)	Cases/PY	IR (95% CI)
**Treatment**							
b/tsDMARD 2nd switchers[1]	8,087	104/10840	0.96 (0.78-1.16)	72/10885.4	0.66 (0.52-0.83)	56/10901.2	0.51 (0.39-0.67)
b/tsDMARD 1st switchers[1]	17,726	155/17841.00	0.87 (0.74-1.02)	107/17863.90	0.60 (0.49-0.72)	83/17887.7	0.46 (0.37-0.58)
First b/tsDMARD switchers users	37,993	364/54138.1	0.67 (0.61-0.75)	263/54215.9	0.49 (0.43-0.55)	165/54297.2	0.30 (0.26-0.35)
csDMARDs	92,509	702/83623.8	0.84 (0.78-0.9)	509/83763.5	0.61 (0.56-0.66)	316/83901	0.38 (0.34-0.42)

1. b/tsDMARD 1st switchers: (1) Define the next switch date. (2) Redefine the censor date by including the switch date. (3) Refine outcome using data from index date to the censor date (earlier date of event date or censor date). (4) Recalculate the rates.

b/tsDMARD 2nd switchers: (1) Check the continuous enrolment and age. (2) Among those eligible, redefine the censor date by including the 3rd switch date. (3) Refine outcome using data from the 2nd switch date (new index date) to the censor date (earlier date of event date or censor date). (4) Recalculate the rates.

Liang H, *et al. RMD Open* 2019; 5:e001013. doi: 10.1136/rmdopen-2019-001013

Supplemental Table S7– Hazard ratios and 95% confidence intervals from multivariate Cox proportional hazard models for venous thromboembolism in patients with rheumatoid arthritis on systemic therapy

Variables in the model	VTE	DVT	PE
b/tsDMARD 2nd vs 1st switchers	1.10 (0.87, 1.38)	1.06 (0.80, 1.40)	1.18 (0.85, 1.62)
b/tsDMARD 2nd switchers vs 1st users	1.48 (1.19, 1.85)	1.42 (1.09, 1.84)	1.74 (1.29, 2.36)
b/tsDMARD 1st switchers vs 1st users	1.35 (1.15, 1.60)	1.34 (1.10, 1.63)	1.48 (1.17, 1.88)
b/tsDMARD 2nd switchers vs csDMARDs	1.62 (1.31, 2.00)	1.55 (1.21, 1.99)	1.86 (1.39, 2.49)
b/tsDMARD 1st switchers vs csDMARDs	1.48 (1.27, 1.72)	1.46 (1.22, 1.76)	1.58 (1.27, 1.98)
First b/tsDMARD users vs csDMARDs	1.09 (0.96, 1.24)	1.09 (0.94, 1.27)	1.07 (0.88, 1.3)
Age 45-64 vs 18-44	1.55 (1.26, 1.91)	1.34 (1.06, 1.7)	1.67 (1.23, 2.27)
Age 65-74 vs 18-44	3.30 (2.66, 4.08)	2.84 (2.23, 3.62)	3.42 (2.49, 4.69)
Age 75+ vs 18-44	4.36 (3.49, 5.44)	4.05 (3.16, 5.2)	4.13 (2.96, 5.75)
Sex (Male vs Female)	1.20 (1.07, 1.35)	1.17 (1.01, 1.34)	1.21 (1.02, 1.43)
Black vs White	1.23 (1.04, 1.46)	1.18 (0.96, 1.46)	1.18 (0.91, 1.52)
Asian vs White	0.64 (0.41, 0.98)	0.46 (0.25, 0.83)	0.78 (0.44, 1.39)
Hispanic vs White	0.73 (0.6, 0.89)	0.75 (0.6, 0.95)	0.63 (0.46, 0.86)
Unknown race vs White	0.80 (0.65, 0.99)	0.82 (0.64, 1.05)	0.85 (0.63, 1.15)

Abbreviation: Cases=number of patients with an outcome event; N=size of the patient population; HR=hazard ratio; CI=confidence interval; VTE= venous thromboembolism; DVT=deep vein thrombosis; PE=pulmonary embolism

Supplemental Table S8– Hazard ratios and 95% confidence intervals from multivariate Cox proportional hazard models for venous thromboembolism in patients with rheumatoid arthritis on systemic therapy who did not have a history of VTE

Variables in the model	VTE	DVT	PE
b/tsDMARD 2nd vs 1st switchers	1.14 (0.88, 1.47)	1.10 (0.80, 1.50)	1.18 (0.83, 1.67)
b/tsDMARD 2nd switchers vs 1st users	1.59 (1.25, 2.02)	1.48 (1.11, 1.99)	1.91 (1.37, 2.67)
b/tsDMARD 1st switchers vs 1st users	1.40 (1.16, 1.68)	1.35 (1.09, 1.69)	1.62 (1.25, 2.11)
b/tsDMARD 2nd switchers vs csDMARDs	1.72 (1.36, 2.17)	1.64 (1.24, 2.17)	1.95 (1.42, 2.68)
b/tsDMARD 1st switchers vs csDMARDs	1.51 (1.27, 1.8)	1.50 (1.22, 1.84)	1.66 (1.3, 2.11)
First b/tsDMARD users vs csDMARDs	1.08 (0.93, 1.25)	1.11 (0.93, 1.32)	1.02 (0.82, 1.27)
Age 45-64 vs 18-44	1.65 (1.3, 2.08)	1.49 (1.14, 1.96)	1.55 (1.12, 2.16)
Age 65-74 vs 18-44	3.30 (2.58, 4.21)	2.90 (2.18, 3.84)	3.20 (2.28, 4.5)
Age 75+ vs 18-44	4.56 (3.54, 5.87)	4.57 (3.42, 6.1)	3.84 (2.68, 5.51)
Sex (Male vs Female)	1.22 (1.07, 1.39)	1.17 (1, 1.37)	1.24 (1.03, 1.5)
Black vs White	1.21 (1, 1.48)	1.15 (0.91, 1.46)	1.21 (0.91, 1.6)
Asian vs White	0.65 (0.4, 1.06)	0.53 (0.28, 0.99)	0.63 (0.31, 1.27)
Hispanic vs White	0.66 (0.52, 0.83)	0.67 (0.51, 0.88)	0.53 (0.37, 0.77)
Unknown race vs White	0.82 (0.65, 1.04)	0.84 (0.64, 1.11)	0.91 (0.65, 1.26)

Abbreviation: Cases=number of patients with an outcome event; N=size of the patient population; HR=hazard ratio; CI=confidence interval; VTE= venous thromboembolism; DVT=deep vein thrombosis; PE=pulmonary embolism

Liang H, *et al. RMD Open* 2019; 5:e001013. doi: 10.1136/rmdopen-2019-001013

To provide a context for the VTE data in RA population, a random sample of 1 million patients without RA in Optum during the study period were retrieved. for screening for eligibility. Their index date was randomly chosen from their clinical visit dates during 2007-2017. To be eligible, they must have at least one year of continuous health plan enrollment before the index date (with a gap of ≤45 days allowed) and at least one day after index date and were at least 18 years old. After applying the inclusion criteria, 322,566 patients without RA were included in the analysis, with mean age 51.1 ± 19.2 (women 53.3%). The crude incidence rate of VTE in general population without RA was 0.41 (0.40-0.43) per 100 patient-years, with age and sex standardized rate being 0.31 (0.30-0.33) per 100 patient-years, see Supplemental figure for the patient attrition and Supplemental Table for age and sex specific rate for VTE presented below.

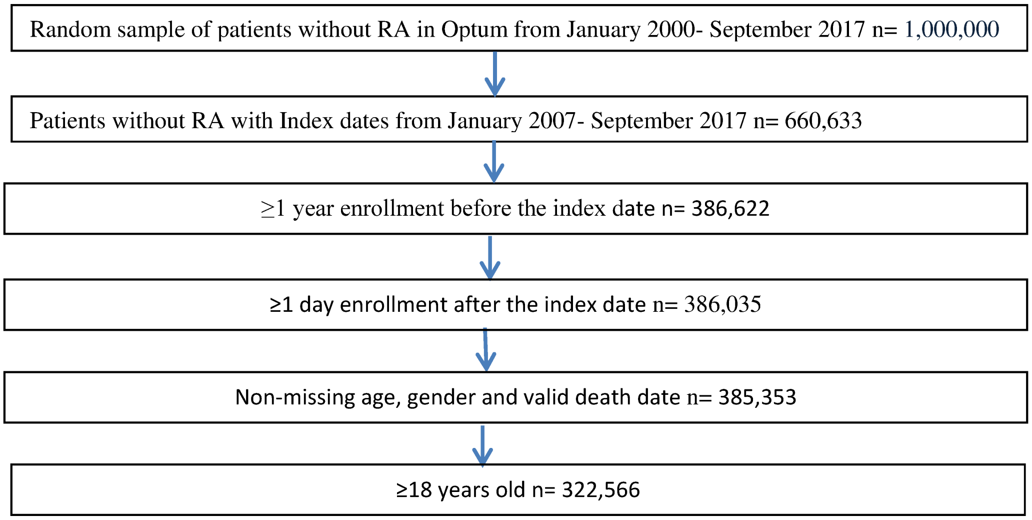

Figure S Attrition of patients without rheumatoid arthritis in Optum 2000-2017

Supplemental Table Age and sex specific incidence rate in the general population without rheumatoid arthritis

	N	Event	Patient-years	Crude IR (95% CI)	Age and sex standardized rate (95% CI)
Male					
18-44	61,236	88	10,4682.7	0.08 (0.07-0.1)	NA
45-64	51,914	382	95,873.6	0.40 (0.36-0.44)	NA
65-74	19,208	322	39,028.5	0.83 (0.74-0.92)	NA
75+	18,337	408	36,968.9	1.1 (1-1.22)	NA
Male total	150,695	1200	276,553.6	0.43 (0.41-0.46)	NA
Female					
18-44	67,854	137	111,451.9	0.12 (0.1-0.15)	NA
45-64	54,391	307	102,193.9	0.30 (0.27-0.34)	NA
65-74	22,629	259	49,432.4	0.52 (0.46-0.59)	NA
75+	26,997	575	57,994.9	0.99 (0.91-1.08)	NA
Female total	171,871	1,278	321,073.1	0.40 (0.38-0.42)	NA
Total	322,566	2478	597,626.7	0.41 (0.40-0.43)	0.31 (0.30-0.33)

Age and Sex-standardized incidence rate was calculated by applying age- and sex-specific incidence rates (i.e. 18-44, 45-64, 65-74, and 75+ for men and women) to population age and sex distribution from US Census 2010 in a direct method[45], with 95% confidence intervals estimated using normal approximation
